# Ethyl Cellulose-Coated Cylindrical Granules for Multi-Unit Sustained-Release Tablets: Trade-Off Between Coating Integrity, Compressibility, Tabletability, and Drug Release

**DOI:** 10.3390/pharmaceutics18101220

**Published:** 2026-09-26

**Authors:** Zhaohua Li, Ying Fan, Jingxi Yang, Guangshuai Zhang, Qiang Fu

**Affiliations:** 1 Wuya College of Innovation, Shenyang Pharmaceutical University, No. 103, Wenhua Road, Shenyang 110016, China; leezh0125spu@163.com (Z.L.); fannying39@163.com (Y.F.); yang_yuduobudoubuy@163.com (J.Y.); zhangguangshuai5@163.com (G.Z.); 2 Joint International Research Laboratory of Intelligent Drug Delivery Systems, Ministry of Education, Shenyang 110016, China; 3 NMPA Key Laboratory for Research and Evaluation of Pharmaceutical Preparations and Excipients, China Pharmaceutical University, Nanjing 210009, China

**Keywords:** cylindrical granules, multi-unit sustained-release tablets, coating film, tableting process, ethyl cellulose

## Abstract

**Background**: Cylindrical granules, rather than spherical pellets, are used in certain multi-unit sustained-release tablets (MUSTs), such as mesalazine (MSZ) sustained-release tablets (Pentasa^®^). Our previous studies established that porosity and aspect ratio govern the tableting behavior of uncoated cylindrical MSZ granules. However, it is unknown how the compressibility, tabletability and release behavior of the coated cylindrical granules will change. **Methods**: In this study, ethyl cellulose (EC) was used as a model sustained-release coating polymer to systematically investigate the effects of EC coating film on the compressibility, tabletability, and drug-release performance of MSZ cylindrical granules. **Results**: It was found that increasing EC molecular weight and coating weight gain compromised the compressibility of the coated cylindrical granules, as reflected by increased yield stress values. Structural analysis demonstrated that thicker coatings and higher-molecular-weight EC films were less susceptible to compression-induced damage, preserving coating integrity after tableting. However, this enhanced coating robustness was accompanied by reduced tabletability, as reflected by lower tablet tensile strength at a given compaction pressure. In addition, increasing EC molecular weight and coating weight gain progressively reduced MSZ release from both coated granules and corresponding tablets. **Conclusions**: These findings demonstrate a trade-off among coating integrity, compressibility, tabletability, and drug-release control in MUST development. The results provide a mechanistic basis and practical guidance for optimizing coated cylindrical granules and balancing coating protection with tablet manufacturability and sustained-release performance.

## 1. Introduction

Oral sustained-release drug delivery systems have gained considerable attention due to their ability to maintain therapeutic drug levels over extended periods [1,2]. Generally, this system is categorized into single-unit dosage forms (SUDFs) and multiple-unit dosage forms (MUDFs) [3]. Compared with SUDFs, MUDFs can disperse more uniformly throughout the gastrointestinal tract [4,5], reducing their susceptibility to dose dumping and minimizing the influence of variability in gastric emptying on drug release [6]. Consequently, MUDFs may provide more consistent drug release and reduce fluctuations in drug exposure and associated adverse effects [7], and have thus been widely adopted in the development of sustained-release formulations.

MUDFs are typically presented as capsules or tablets containing coated granules or pellets. Compared with capsule-based systems, tablet-based MUDFs offer lower production costs, greater manufacturing efficiency, and improved patient convenience [8,9,10]. However, the tableting process poses considerable challenges to the quality of multi-unit sustained-release tablets (MUSTs). On one hand, compaction pressure can disrupt the coating film on granules, thereby compromising the sustained-release function [11]. Extensive studies have therefore investigated the effects of coating materials [12,13], cushioning excipients, and tableting parameters [1,14] on the release profile of MUSTs. On the other hand, coating inevitably changes the physicochemical and mechanical properties of the granules, including their morphology, specific surface area, interparticle interactions, and mechanical strength. These changes may substantially affect granule deformation, densification, compressibility, and tabletability, thereby influencing the manufacturability of MUSTs.

Most compression-coating studies have used spherical granules or pellets, in which interparticle contacts are essentially point contacts, and the coating film experiences relatively uniform stresses during tableting. However, cylindrical granules differ fundamentally in packing, contact mechanics, and deformation. They tend to align and interlock, forming line or face contacts rather than point contacts. Therefore, the stress distribution, film bending, and compression-induced damage in cylindrical granules cannot be inferred from spherical pellet systems. Our previous studies (Parts I and II) established the tableting behavior of uncoated cylindrical mesalazine granules, showing that porosity and aspect ratio govern their compressibility and tabletability [15,16]. Whether these geometry-related characteristics are preserved after coating remains unknown. This study therefore focuses on film-coated cylindrical granules, using the molecular weight and coating weight gain of coating materials as tools to vary coating-film mechanical strength and further study their influences on the tableting process.

Ethyl cellulose (EC), a hydrophobic cellulose derivative, possesses excellent film-forming properties and is frequently employed as a coating material for MUSTs [17]. The physicochemical properties of EC, such as molecular weight (M.W.), viscosity, and dosage, can substantially influence the release profiles of MUSTs [13,18,19,20]. However, the specific impact of EC coating on the tableting process, as distinct from its impact on drug release, has not been systematically explored. Mesalazine (MSZ) sustained-release tablets (Pentasa^®^) are representative MUSTs compressed from EC-coated MSZ cylindrical granules [15,16] and are widely used for the treatment of inflammatory bowel disease [21,22]. Therefore, this formulation was selected as the model system for the present study.

Building upon our previous findings on uncoated cylindrical granules [15,16], the present study investigated how EC coating influences the compression behavior, tabletability, coating integrity, and drug release of MSZ cylindrical granules and their corresponding MUSTs. EC was selected as a model brittle sustained-release coating polymer, and EC molecular weight and coating weight gain were used as controlled variables. Specifically, the viscosities of EC grades with different molecular weights were first determined. MSZ cylindrical granules were then coated with different EC grades at various coating weight gains using a fluidized bed. The size, density, and hardness of the EC-coated granules were characterized. Their compressibility and tabletability were evaluated using mathematical models and scanning electron microscopy (SEM). Finally, release tests were conducted to compare the release profiles of EC-coated granules and their corresponding tablets. By elucidating the effects of EC coating on granule structure, coating film integrity, compaction behavior, and drug release, this study provides mechanistic insight and practical guidance for the rational development of EC-coated cylindrical granules and MUSTs.

## 2. Materials and Methods

### 2.1. Materials

MSZ was purchased from Zhejiang Hengkang Pharmaceutical Co., Ltd. (Taizhou, China). EC was gifted by Ashland Investment Co., Ltd. (Shanghai, China). Magnesium stearate was provided by Ron Pharm Co., Ltd. (Shanghai, China). All other reagents and chemicals were of analytical grade.

### 2.2. Determination of EC Viscosity

The dynamic viscosity of the EC solution was measured using a DV2T rotational viscometer (Brookfield, Middleboro, MA, USA) equipped with a No. 62 spindle. Firstly, 5.0 g of EC was accurately weighted and placed in a stoppered conical flask containing 95 g of ethanol–toluene solution (1:4, *w*/*w*). Then, the flask was shaken until the EC was fully dissolved. Finally, the dynamic viscosity of the EC solution was determined. The rotation speed of the spindle was set at 50 rpm, and the test temperature was maintained at 25 °C.

### 2.3. Preparation of EC-Coated Cylindrical Granules

As described in our previous study [15,16], MSZ cylindrical granules were extruded using an XL-120 basket radial extruder (Changzhou Jiafa Granulation and Drying Equipment Co., Ltd., Changzhou, China), and their sizes were controlled over the range of 800–1000 μm by sieving.

Cylindrical granules (400 g) were then coated by spraying a 3% (*w*/*w*) EC acetone solution using a FZBL 1.5 fluidized bed (Chanse Technology Inc., Changzhou, China). Coated cylindrical granules with different coating weights were obtained by controlling the amount of coating solution sprayed. The following coating parameters were kept throughout the preparation process: preheat for 5 min before spraying; inlet temperature 40–45 °C; bed temperature 35–40 °C; air flow 110–120 m^3^/h; atomization pressure 0.9 bar; feed rate 10 mL/min; and 45 °C bed temperature for 10 min drying after coating. Finally, the sizes of coated cylindrical granules were controlled over the range of 800–1180 μm by sieving.

### 2.4. Physical Characterizations of EC-Coated Cylindrical Granules

#### 2.4.1. Length and Width

The length and width of coated cylindrical granules were determined using a YS2H optical microscope (Nikon Corporation, Tokyo, Japan). Their aspect ratio (AR) was then calculated according to Equation (1):

(1) AR=l/b

where *l* and *b* represent the length and the width of cylindrical granules, respectively.

#### 2.4.2. Bulk and Tapped Density

The bulk and tapped densities of coated cylindrical granules were measured (*n* = 3) using an Autotap vibratory densimeter (Anton Paar Trading Co., Ltd., Shanghai, China). The Hausner ratio (HR), an indicator of granule flowability, was calculated using Equation (2).

(2) $$\mathrm{HR}=\rho_{t}/\rho_{b}$$

where *ρ_t_* is the tapped density and *ρ_b_* is the bulk density.

#### 2.4.3. Hardness

The hardness of coated cylindrical granules was determined using a CT3 Texture Analyzer (Brookfield, Middleboro, MA, USA). Each cylindrical granule was positioned horizontally before being compressed with a TA5 probe. The trigger force was set to 0.05 N, while the pre-test, test, and post-test speeds were configured at 0.5 mm/s, 0.02 mm/s, and 5.0 mm/s, respectively.

### 2.5. Tableting of Coated Cylindrical Granules

EC-coated cylindrical granules, each containing 500 mg of MSZ, were blended with 1% magnesium stearate and subsequently compacted under pressures ranging from 10 to 300 MPa. A ZP8 rotary tablet press (Shanghai Tianxiang Jiantai Pharmaceutical Machinery Co., Ltd., Shanghai, China) equipped with 10 mm flat-faced punches was used for the compaction process. Following compaction, tablet weight, diameter, and height were carefully measured.

Then, the porosity of tablets was calculated by Equation (3):

(3) $$\varepsilon =1-\frac{4w}{\pi d^{2}h\rho_{true}}$$

where *ε* is the tablets’ porosity; *ρ_true_* is the true density of cylindrical coating granules; and *d*, *h*, and *w* represent the diameter, height, and weight of tablets, respectively. We also added our method for determining the true density in the Supplementary Material.

Subsequently, the breaking force of the MSZ tablets was determined using a YPD-200C tablet hardness tester (Shanghai Huanghai Pharmaceutical Testing Instrument Co., Ltd., Shanghai, China). The tensile strength was then calculated by Equation (4).

(4) $$\sigma =\frac{2F}{\pi dh}$$

where *σ*, *F*, *d*, and *h* represent the tensile strength, diametrical breaking force, diameter, and height of a tablet, respectively.

### 2.6. Compressibility and Tabletability Evaluation

#### 2.6.1. Compressibility Evaluation

The out-of-die Heckel analysis (Equation (5)) was employed to assess the compressibility of coated cylindrical granules [23]. The mean yield pressure (P_y_), serving as an indicator of compressibility, was determined from the reciprocal of the slope of the Heckel plot (Equation (6)). A lower P_y_ value signifies higher compressibility.

(5) $$-ln(\varepsilon )=K_{1}P+A$$

(6) $$P_{y}=1/K_{1}$$

where *P* is the compression pressure, *ε* represents the porosity of tablets at compression, *A* is a constant, and *K*_1_ is the slope of the linear portion of the Heckel curve.

#### 2.6.2. SEM

The morphology of the coated cylindrical granules after compaction (at 40 MPa) was observed using a ZEISS Gemini 300 scanning electron microscope (Carl Zeiss AG, Oberkochen, Germany) at an acceleration voltage of 15 kV. Firstly, MSZ tablets were broken into two halves using the texture analyzer, and the debris and powder on the fracture surface were carefully removed. Then, the fractured tablets were affixed to conductive tape with the fracture surface facing upward. Subsequently, the fracture surface of the tablet was observed under a microscope. The side surfaces of coated cylindrical granules were inspected for signs of damage, wrinkling, or coating detachment, while the bottom or cross-section of them was analyzed to assess the degree of deformation.

#### 2.6.3. Tabletability Evaluation

The tabletability of coated cylindrical granules was evaluated by fitting a Power model to the tensile strength and compression pressure [24] (Equation (7)). High tensile strength represents good tabletability under the same compaction pressure.

(7) $$\sigma =K_{2}P^{g}$$

where *σ* is the tensile strength of tablets, *K*_2_ refers to the constant, and *g* represents the pressure sensitivity index.

### 2.7. In Vitro Release Testing of EC-Coated Cylindrical Granules and Tablets

The release test was conducted using a DS1206 dissolution tester (Shenzhen WAHYONG Technology Co., Ltd., Shenzhen, China). A 900 mL phosphate buffer solution (PBS, pH 6.8) maintained at 37 ± 0.5 °C served as the dissolution medium, with the paddle rotation speed at 50 rpm. Coated cylindrical granules or tablets (containing 500 mg of MSZ) were placed into each vessel (*n* = 3), and approximately 10 mL of solution was sampled at 0.5, 1, 2, 3, 6, 9, 12, 16, and 24 h. Each sample was filtered through a 0.45 μm membrane, and the concentration of MSZ was measured at 330 nm using a T-6 UV spectrophotometer (Nanjing Philes Instrument Co., Ltd., Nanjing, China).

## 3. Results and Discussion

### 3.1. EC Viscosity

According to specifications provided by Asland^TM^, the MWs of EC N7, N22, N50, and N100 are 6500, 140,000, 160,000, and 215,000, respectively. To elucidate the relationship between MW and the viscosity of EC, the dynamic viscosities of all ECs were determined. As shown in Figure 1, the dynamic viscosities of EC N7 and N22 were less than 50 mPa·s, while those of EC N50 and N100 were higher than 100 mPa·s. Thus, the higher-molecular-weight EC grades exhibited substantially greater viscosities than the lower-molecular-weight grades. This behavior can be attributed to the molecular-chain characteristics of EC. An increase in MW corresponds to a greater number of anhydroglucose units and a longer polymer backbone [25]. In solution, longer EC chains occupy a larger hydrodynamic volume and are more prone to chain overlap and entanglement. These effects increase intermolecular friction and restrict polymer-chain mobility, thereby increasing the resistance of the solution to flow [25,26]. The viscosity of the EC coating solution is an important process parameter in fluidized-bed coating. Differences in solution viscosity may affect not only the coating efficiency and film morphology but also the mechanical response of the coating layer during subsequent compaction. These potential effects provide a basis for further examining the relationship between EC molecular weight, coated-granule properties, tabletability, and coating-film integrity.

### 3.2. Physical Characterizations of EC-Coated Cylindrical Granules

To study the influences of the coating process on MSZ cylindrical granules, physical properties (size, AR, density, and flowability) of EC-coated granules were characterized. As shown in Table 1, the length of the coated cylindrical granules in each group ranged from 1800 to 1900 μm, the width ranged from 900 to 1000 μm, and AR values were between 1.9 and 2.1. In addition, there are no significant differences among these groups (*p* > 0.05), indicating that the particle sizes were homogeneous. Thus, the coated granules retained the geometric characteristics of the uncoated cores, allowing the effects of coating weight gain and EC molecular weight to be evaluated without major confounding effects arising from changes in particle size or AR.

Moreover, with the increase in coating weight, both the bulk density and tapped density of the coated cylindrical particles slightly increased. This increase was likely attributable to the deposition of EC over the intergranular surface region, which increased the mass of the granules and may have reduced accessible surface irregularities or near-surface voids. In addition, the Hausner ratio of all granules did not exceed 1.07, indicating their good flowability.

Additionally, hardness values of all coated granules were determined. As shown in Figure 2, under the same coating weight gain, the higher the MW of EC, the greater the hardness of the corresponding granules. This is because EC with higher MW contains longer polymer chains with a greater capacity for chain overlap and entanglement, as well as stronger interchain interactions [18]. After solvent evaporation, these characteristics may generate a more cohesive and mechanically resistant polymer network, thereby improving the hardness of the coated granules [18].

### 3.3. Compressibility Evaluation

To identify the role of EC coating film on the tableting process of granules, the compressibility of all EC-coated cylindrical granules was evaluated using Heckel analysis. The results are shown in Figure 3. *P_y_* values of coated cylindrical granules increased as EC MW and coating weight gain increased, indicating that the coated granules exhibited greater resistance to plastic deformation under compression; that is, the compressibility was reduced. For example, when the coating weight gain increased from 2% to 15%, the *P_y_* values of N7-coated cylindrical granules increased from 35.71 to 50.25, while that of N100-coated cylindrical granules increased from 46.51 to 66.20.

### 3.4. SEM

To further elucidate the influences of MW and weight gain on the granules, the morphology of the coated cylindrical granules after compression was observed using SEM. As shown in Figure 4, granules coated with low-MW EC (N7 and N22) and with low coating weight gain (2% and 5%) exhibited considerable damage and peeling after compression. In contrast, for cylindrical granules coated with high-MW EC (N50 and N100) and with high coating weight gain (10% and 15%), the surface coating film showed almost no damage, with only slight deformation and shrinkage. These findings suggest that increasing the EC MW and coating weight gain improved the integrity of the coating film during compression.

The cross-sectional morphologies shown in Figure 5 provide further evidence of this behavior. When the EC MW and coating weight gain were low, the cross-section of the cylindrical granules was compressed into an elliptical shape, with a significant degree of deformation. By contrast, granules prepared with higher-MW EC and higher coating weight gains retained an approximately circular cross-sectional shape. The preservation of the original cross-sectional geometry indicates that the stronger coating films resisted radial deformation during compression.

In Section 3.2, we confirmed that the hardness of coated granules was directly proportional to the MW (i.e., the viscosity) of EC. Therefore, MSZ cylindrical granules coated with high-MW EC were difficult to compress and thereby showed low compressibility. This interpretation is consistent with the findings of Upadrashta et al., who evaluated the compressibility of pure EC powder using Heckel analysis. They reported that the *P_y_* value of pure EC powder would increase from 66.2 to 104.8 MPa as their viscosities increased from 10 to 100 mPa·s [27], indicating the enhancement of anti-compression ability. Although the experimental systems differed from those used in the present study, their results provide indirect support for the conclusion that the viscosity-related mechanical properties of EC can substantially influence the compressibility of EC-coated granules.

### 3.5. Tabletability Evaluation

The tabletability of the EC-coated granules was evaluated by examining the relationship between compaction pressure and tablet tensile strength. As shown in Figure 6, at a given compaction pressure, an increase in the coating weight for the same MW of EC resulted in a decrease in the tensile strength of tablets. For example, a tablet compacted from cylindrical granules with an EC N7 coating film (weight gain = 2%) had a tensile strength of 5.53 MPa. However, when coating weight gain increased to 15%, the tensile strength of the corresponding tablet was only 2.97 MPa. Similarly, for the same coating weight, tablets prepared from granules coated with higher-MW EC generally exhibited lower tensile strengths. For instance, a 10% N7 coating corresponds to a tablet tensile strength of 4.44 MPa, whereas a 10% N100 coating results in a tensile strength of only 2.31 MPa. These results demonstrate that increases in both EC MW and coating weight gain reduced the tabletability of the coated cylindrical granules.

The bonding area–bonding strength (BA–BS) model proposed by Sun et al. provides a mechanistic framework for interpreting the tableting behavior of powders and granules [28]. According to this model, tablet tensile strength is governed by the combined contributions of BA and BS. BA represents the extent of interparticulate contact formed during compaction, whereas BS describes the strength of bonding per unit contact area. Therefore, the formation of tablets with high tensile strength generally requires a sufficiently large BA, a high BS, or both [29].

Usually, BA varies with particle size, shape, and deformation degree [28]. In our study, the length, width, and AR of the EC-coated granules did not differ significantly among the formulations. Therefore, the differences in tabletability were unlikely to have resulted primarily from variations in the initial size or shape of the granules. Instead, differences in their deformation behavior were likely a major determinant of BA. As demonstrated by the Heckel and SEM results, granules coated with high-MW EC had worse compressibility than those coated with low-MW EC. The limited deformation of these mechanically stronger granules restricted the development of intergranular contact areas during compaction, thereby producing a smaller BA and tablets with lower tensile strength. Increasing the coating weight gain may have reduced BA through a similar mechanism. A thicker or more continuous EC layer provided greater mechanical reinforcement to the granules, limiting their deformation during compression. Although this improved the structural integrity of the individual coated granules, it reduced their ability to flatten and conform to neighboring particles. Consequently, fewer or smaller effective contact areas were generated between adjacent granules, resulting in weaker tablet structures.

BS is dependent on interactions among granules. It has been reported that higher-MW EC tended to exhibit stronger elasticity recovery during compression [27]. Elastic recovery can partially separate intergranular contact surfaces formed under pressure, reducing the effective contact area retained after unloading and weakening the resulting intergranular bonds. Moreover, because the outer surfaces of the granules were predominantly covered by EC, tablet formation depended largely on EC–EC interfacial bonding. A thicker coating increased the proportion of these polymer-dominated contacts while reducing the possible contribution of the underlying granule cores to interparticle bonding. The combined effects of limited contact-area development and post-compaction elastic recovery therefore explain the decrease in tablet tensile strength with increasing EC MW and coating weight gain. This interpretation is consistent with previous studies showing that highly elastic materials generally exhibit poor tabletability because substantial elastic recovery can disrupt interparticulate contacts and promote defects such as capping and lamination [30,31,32,33,34]. For example, Shubhajit Paul et al. investigated the tableting behavior of 26 formulations using machine-learning and multivariate approaches and found that formulations with pronounced elastic behavior had poorer compaction performance and a greater tendency toward capping []. Collectively, these findings support the conclusion that high-MW EC coatings enhanced the mechanical integrity of individual granules but reduced tabletability by limiting BA development and weakening the contacts retained after decompression.

### 3.6. In Vitro Release Testing of Coated Cylindrical Granules

As shown in Figure 7, as the MW of EC increased, release rates of both coated cylindrical granules and the corresponding tablets decreased. This behavior may be attributed to differences in the structure and integrity of the EC coating films. High-MW EC contains longer polymer chains, which promote chain overlap and entanglement and facilitate the formation of a more cohesive coating network with lower permeability. Consequently, the penetration of the release medium into the granules and the outward diffusion of the dissolved drug were hindered, resulting in slower drug release. By contrast, films formed from low-MW EC may have exhibited lower cohesiveness and greater permeability, thereby providing less resistance to drug diffusion [35].

Additionally, with an increase in coating weight, the release rate also showed a decreasing trend. In particular, when the coating weight increased from 2% to 5%, the cumulative release amount sharply declined. According to Fick’s laws of diffusion [36,37], an increase in the thickness of the coating membrane can extend the distance over which the drug diffuses, thereby reducing the rate of drug exchange between the interior and exterior of the coating [38].

Importantly, the effect of tableting on drug release depended on both EC MW and coating weight gain. When the MW of EC and weight gain were lower, the coated granules showed a higher release rate after being compressed into tablets. However, as MW and weight gain increased, the difference in release behaviors between granules and tablets obviously decreased. This difference was likely caused by compression-induced damage to the coating films. As shown by the SEM observations, the films formed using low-MW EC at low coating weight gains underwent substantial cracking, peeling, or other structural damage during compaction. Such damage may have generated additional pathways for medium penetration and drug diffusion, thereby compromising the sustained-release function of the coating and accelerating drug release from the tablets.

As EC MW and coating weight gain increased, the difference between the release profiles of the coated granules and their corresponding tablets became progressively smaller. High-MW EC and increased coating weight gain enhanced the structural integrity of the coating films. Consequently, fewer compression-induced defects were formed, and the original diffusion-barrier function of the coating was largely retained after compaction. These findings are consistent with the crushing-strength, Heckel, and SEM results, collectively demonstrating that mechanically stronger EC coatings were more effective in preserving the drug-release characteristics of the coated granules during tableting. Thus, although increases in EC MW and coating weight gain reduced granule compressibility and tabletability, they improved the resistance of the coating film to compaction-induced damage and minimized changes in drug release after tableting.

This study was designed as a mechanistic investigation using EC as a model brittle sustained-release coating polymer rather than a comprehensive formulation screening. Additives such as magnesium stearate and talc, plasticizers, and flexible polymers such as Kollidon SR or Eudragit may further modulate coating integrity, compressibility, tabletability, and drug release. These variables were beyond the scope of the present work and will be investigated in future formulation-development studies.

## 4. Conclusions

In summary, this study demonstrates that, for EC-coated mesalazine cylindrical granules and their corresponding MUSTs, increasing EC molecular weight and coating weight gain improve coating integrity against compression-induced damage but reduce compressibility and tabletability, and decrease drug release. These results reveal a trade-off among coating protection, tablet manufacturability, and sustained-release control. EC was used as a model brittle coating polymer; further studies are warranted to evaluate other coating polymers, plasticizers, and additives. The findings provide a mechanistic basis for optimizing EC-coated cylindrical granules and balancing coating integrity with tablet performance.

## Figures and Tables

**Figure 1:**
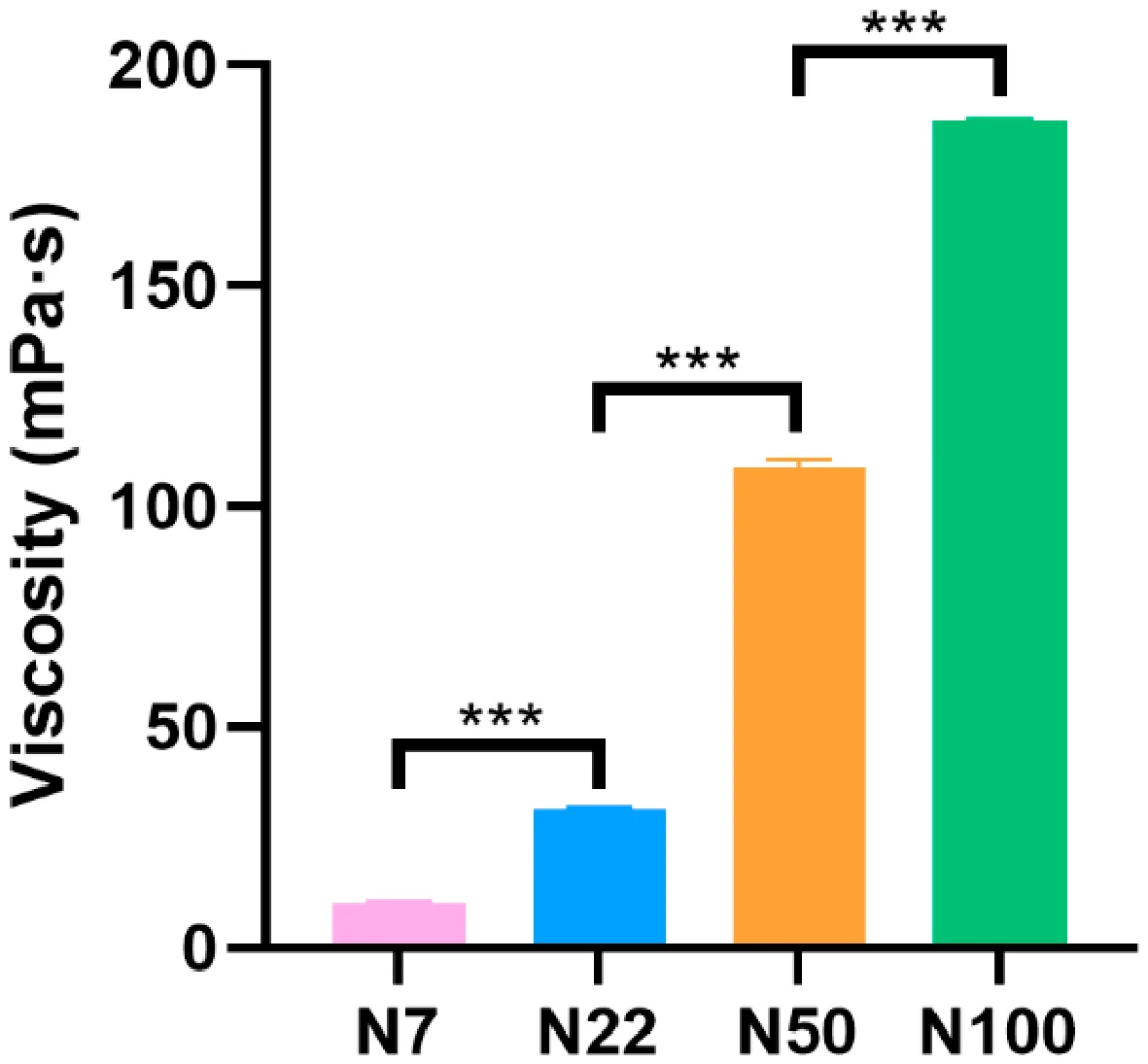
Dynamic viscosities of EC with different molecular weights (*** represents *p* < 0.01).

**Figure 2:**
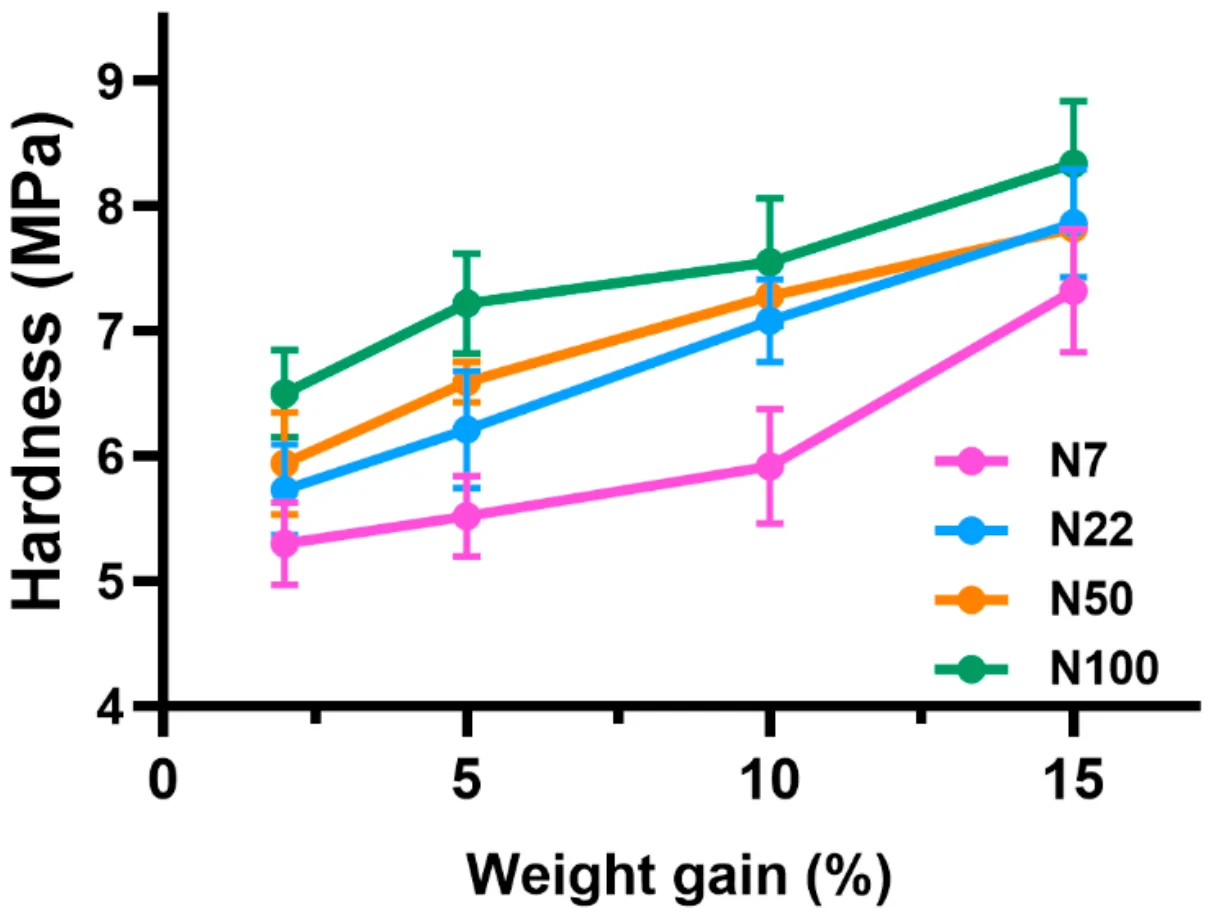
Hardness of MSZ cylindrical granules coated with different EC.

**Figure 3:**
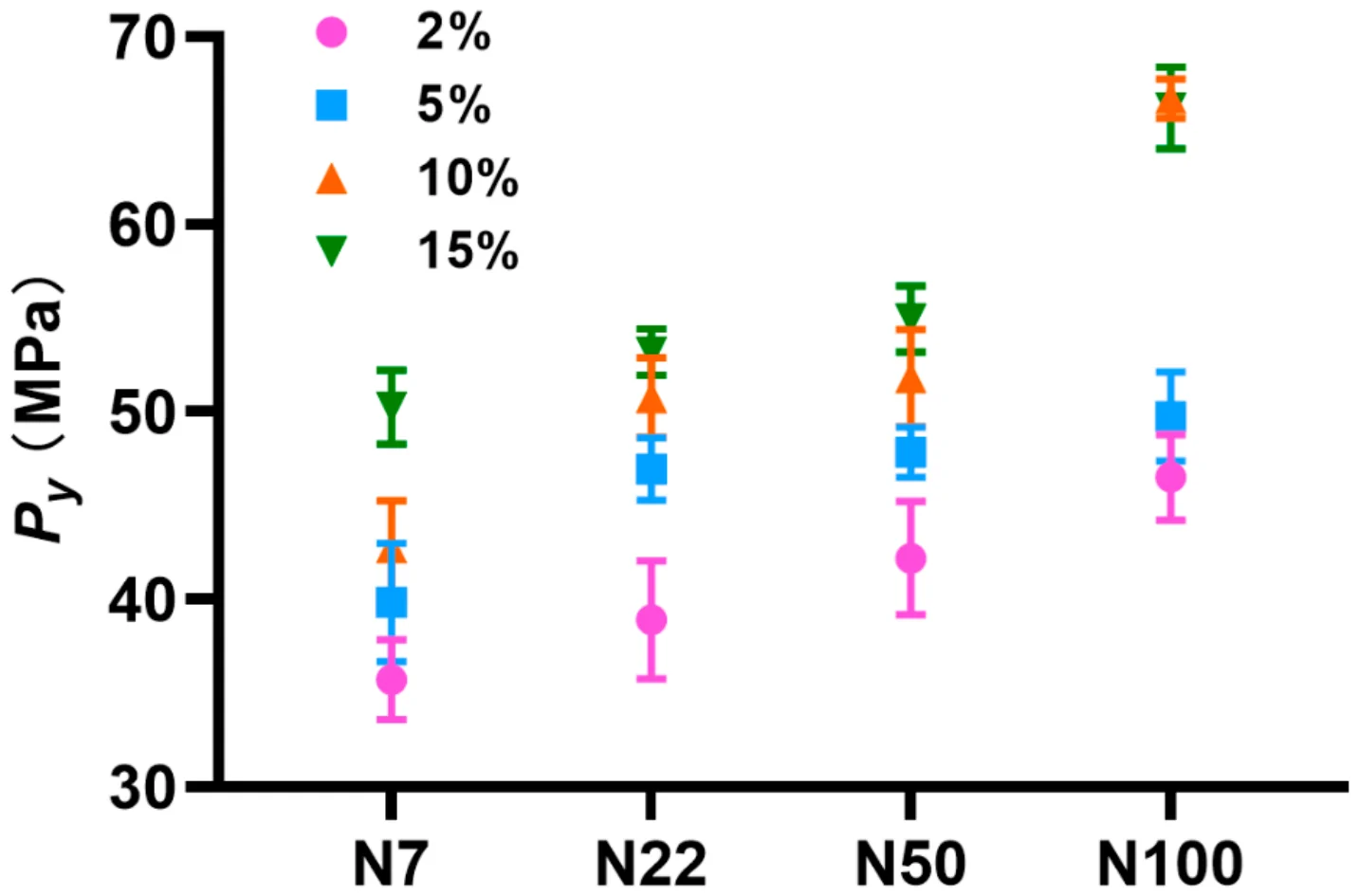
*P_y_* values of MSZ cylindrical granules coated with different EC.

**Figure 4:**
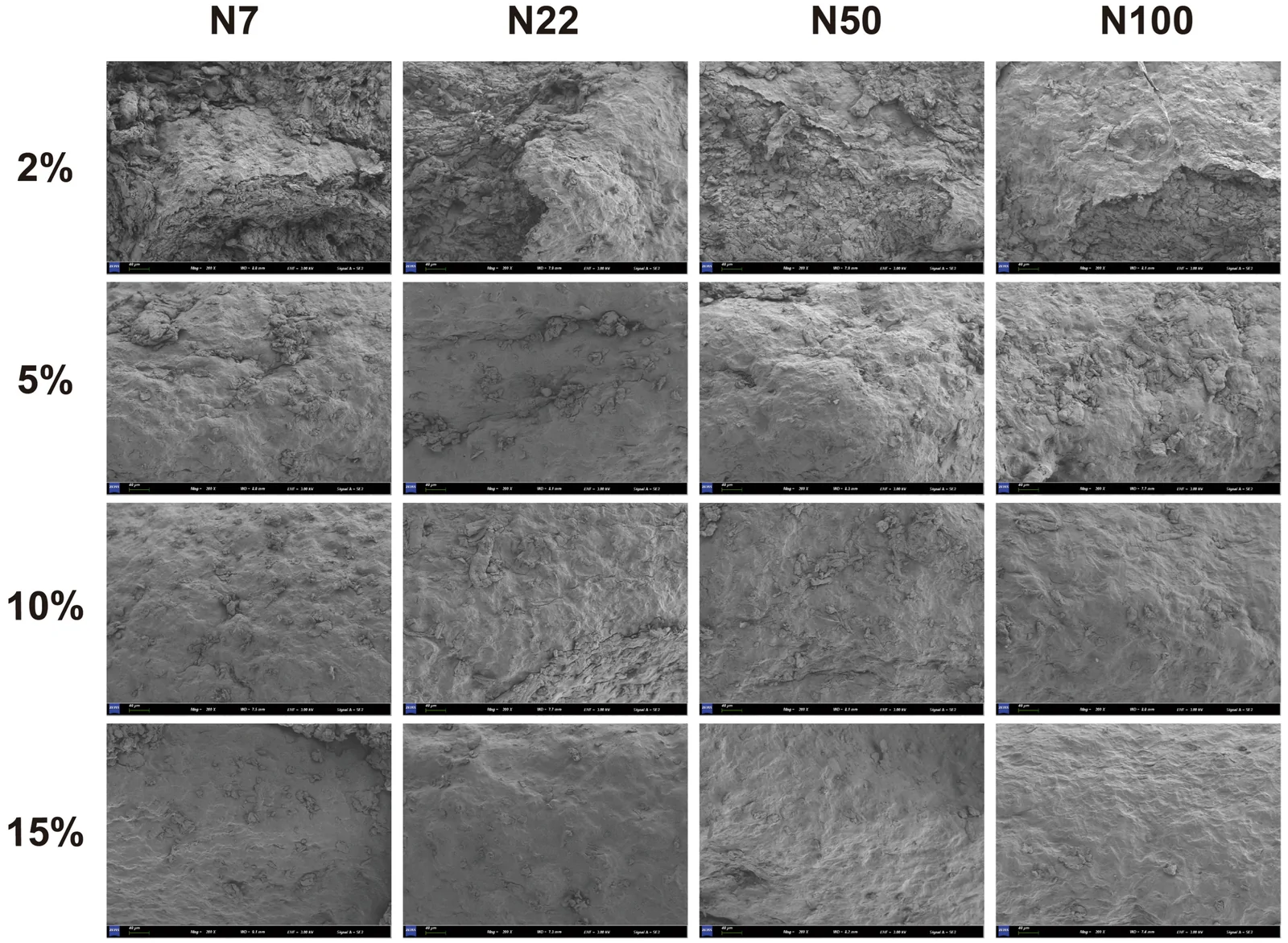
Scanning electron micrographs of the lateral surface of EC-coated cylindrical granules after compression at 40 MPa.

**Figure 5:**
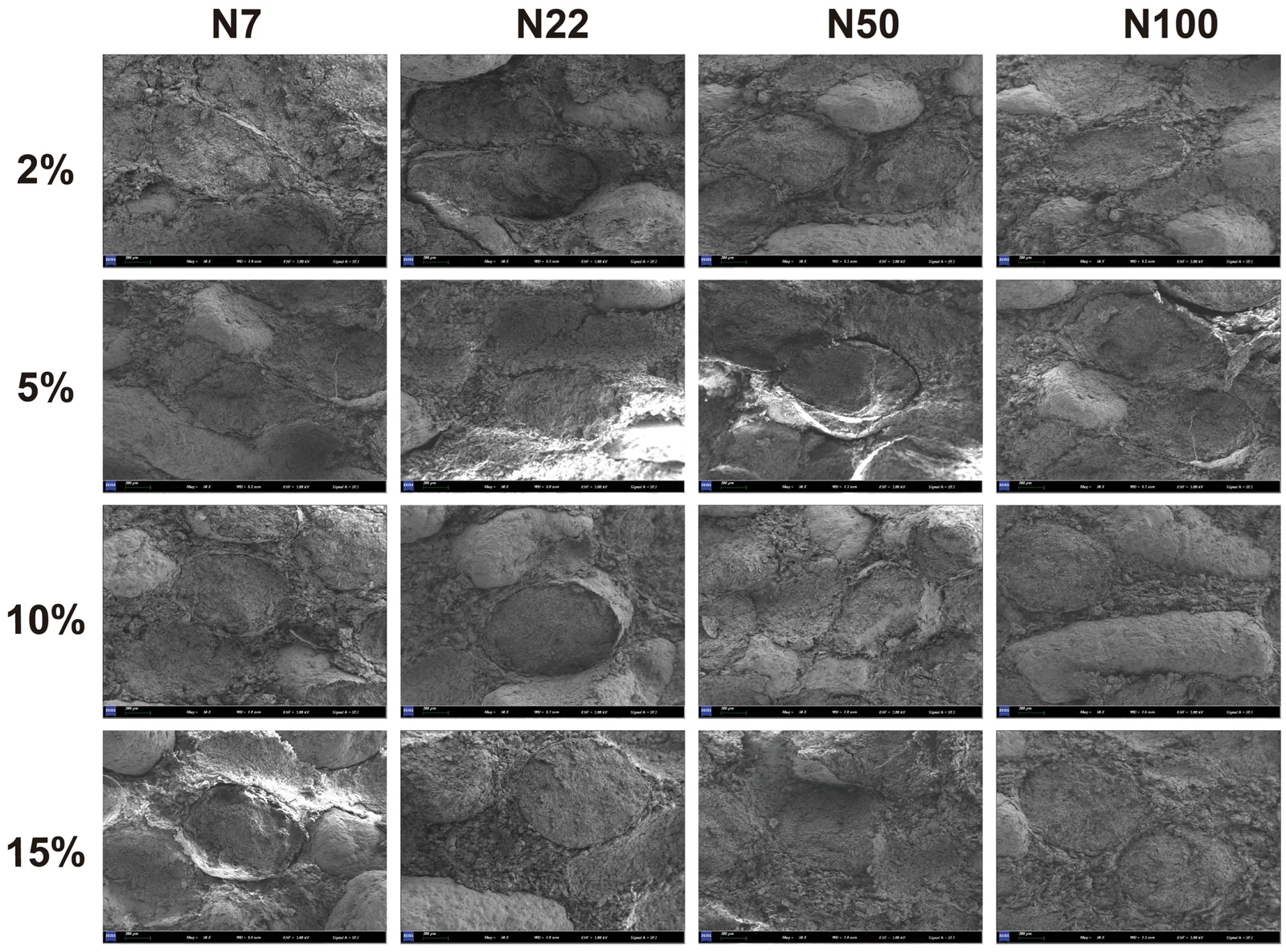
Scanning electron micrographs of the bottom surface of MSZ cylindrical granules coated with different EC after compression at 40 MPa.

**Figure 6:**
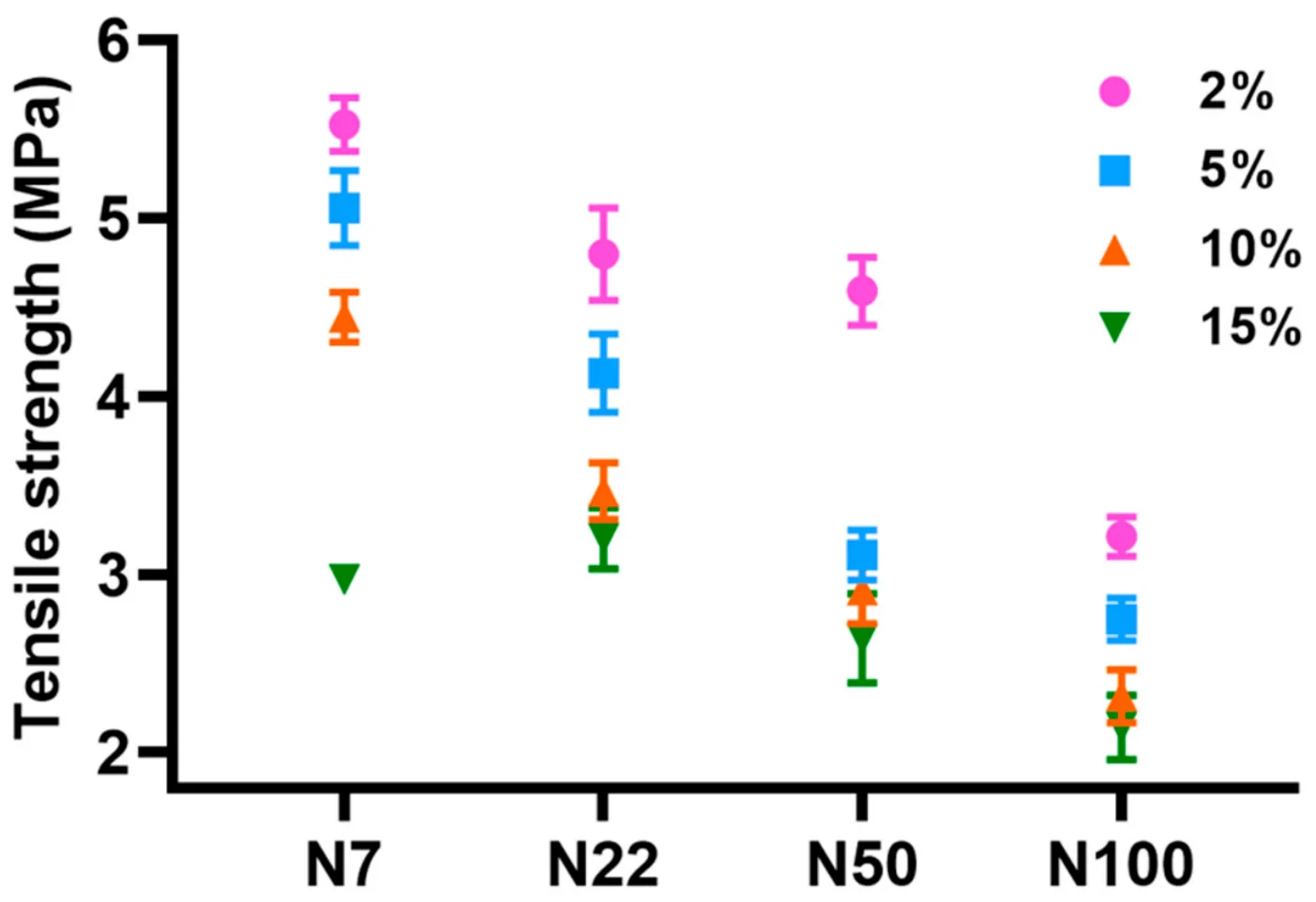
Tensile strength of tablets compressed from MSZ cylindrical granules coated with different EC (compaction pressure: 100 MPa).

**Figure 7:**
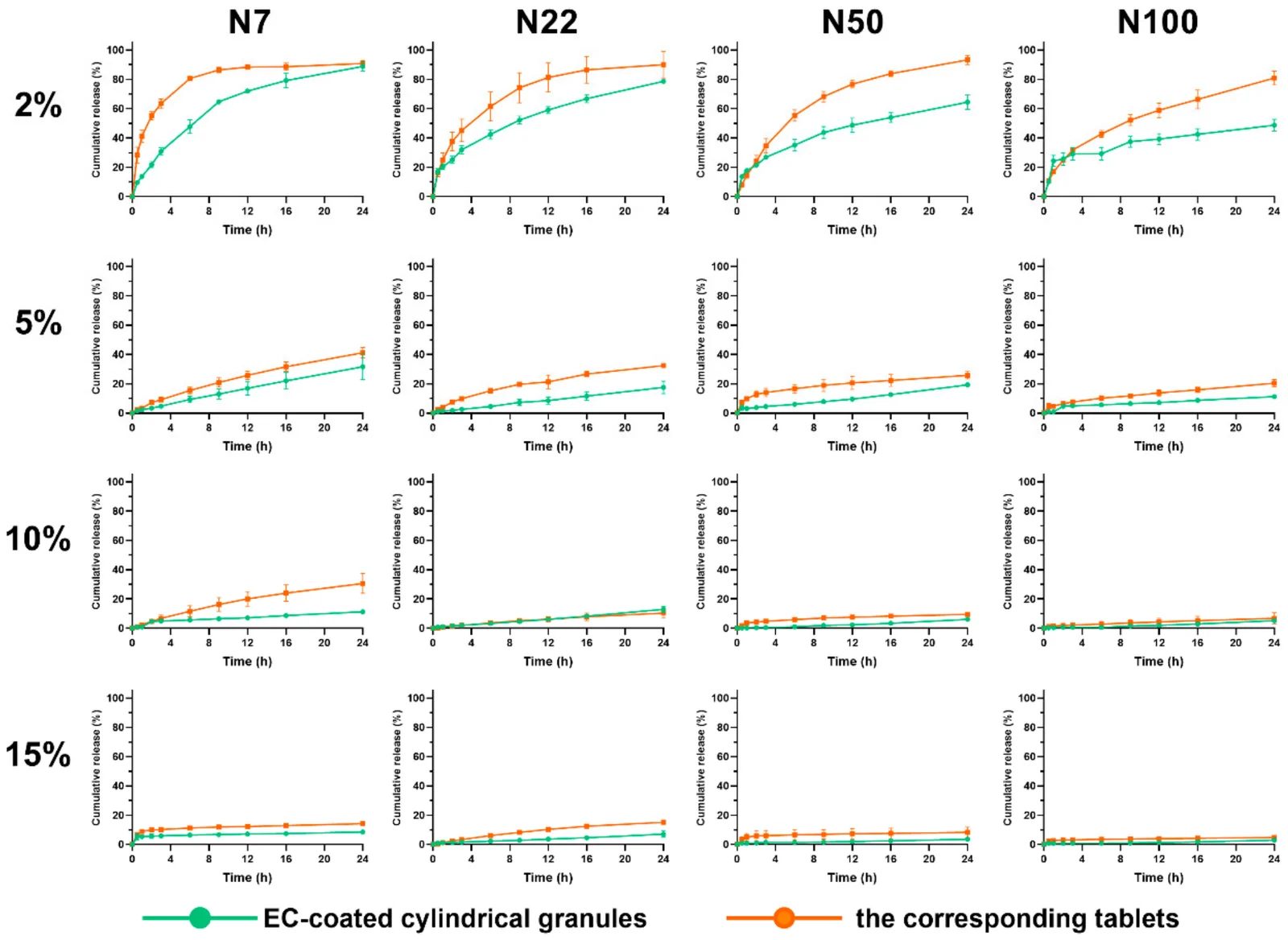
Release profiles of coated MSZ cylindrical granules and their corresponding tablets (compacted at 40 MPa) in pH 6.8 PBS over 24 h.

**Table 1:** Physical properties of MSZ cylindrical granules coated with EC.

EC Type	Weight Gain (%)	Length (μm)	Width (μm)	AR	Bulk Density (g/cm^3^)	Tapped Density (g/cm^3^)	Hausner Ratio
Uncoated	0%	1814 ± 78	937 ± 33	1.94	0.584 ± 0.006	0.604 ± 0.002	1.03
N7	2%	1847 ± 164	962 ±19	1.92	0.597 ± 0.013	0.616 ± 0.014	1.03
	5%	1847 ± 119	951 ± 29	1.94	0.608 ± 0.009	0.630 ± 0.008	1.04
	10%	1854 ± 101	911 ± 42	2.04	0.623 ± 0.002	0.641 ± 0.005	1.03
	15%	1862 ± 136	917 ± 121	2.03	0.630 ± 0.002	0.644 ± 0.001	1.02
N22	2%	1806 ± 154	922 ± 27	1.96	0.580 ± 0.001	0.599 ± 0.001	1.03
	5%	1860 ± 85	927 ± 67	2.01	0.593 ± 0.005	0.617 ± 0.013	1.04
	10%	1850 ± 55	905 ± 37	2.04	0.598 ± 0.001	0.625 ± 0.001	1.05
	15%	1854 ± 156	966 ± 63	1.92	0.622 ± 0.003	0.635 ± 0.002	1.02
N50	2%	1806 ± 109	928 ± 19	1.95	0.581 ± 0.001	0.614 ± 0.001	1.06
	5%	1824 ± 103	930 ± 44	1.96	0.604 ± 0.003	0.634 ± 0.002	1.05
	10%	1842 ± 113	920 ± 31	2.00	0.617 ± 0.005	0.631 ± 0.013	1.02
	15%	1864 ± 177	950 ± 27	1.96	0.625 ± 0.006	0.644 ± 0.005	1.03
N100	2%	1867 ± 115	925 ± 56	2.02	0.572 ± 0.006	0.614 ± 0.003	1.07
	5%	1874 ± 63	914 ± 40	2.05	0.612 ± 0.002	0.629 ± 0.001	1.03
	10%	1891 ± 80	937 ± 23	2.02	0.621 ± 0.001	0.630 ± 0.002	1.01
	15%	1898 ± 113	961 ± 48	1.98	0.623 ± 0.001	0.645 ± 0.001	1.04

## Data Availability

Data will be made available on request.

## Supplementary Materials

The following supporting information can be downloaded at: https://www.mdpi.com/article/10.3390/pharmaceutics18101220/s1. Reference [39] is cited in the Supplementary Materials.
