# Supplementary material for: Ethyl Cellulose-Coated Cylindrical Granules for Multi-Unit Sustained-Release Tablets: Trade-Off Between Coating Integrity, Compressibility, Tabletability, and Drug Release
Source: Pharmaceutics. 2026 Sep 26;18(10):1220. doi: 10.3390/pharmaceutics18101220 (PMC13708593; doi:10.3390/pharmaceutics18101220)
Supplement: Supplementary file 1 [file pharmaceutics-18-01220-s001.zip › pharmaceutics-4549553-supplementary.pdf]

Determination of true density

MSZ cylindrical granules (200 mg) were compressed (10–300 MPa) using a ZP8 instrumented rotary tablet press (Shanghai Tianxiang Chentai Pharmaceutical Machinery Co., Ltd., Shanghai, China) equipped with 8.0 mm flat-faced punches. The tableting speed was set at 15 rpm and the punch was lubricated with 1 % magnesium stearate ethanol suspension to avoid tablet sticking. After compression, the weight, diameter, and height of the tablets were measured. The tablet density was then calculated using Eq. (1).

$$\rho = \frac{4w}{\pi d^2 h} \qquad (1)$$

in which the ρ, d, h, and w are the density, diameter, height, and weight of tablets, respectively.

Finally, as reported in the previous study [1, 2], the true density was obtained by the nonlinear fitting of the pressure and the tablet's density.

$$P = \frac{1}{C}\left\{(1 - \varepsilon_C) - \frac{\rho}{\rho_{ture}} - \varepsilon_c \ln\left(\frac{1 - \frac{\rho}{\rho_{ture}}}{\varepsilon_c}\right)\right\} \qquad (2)$$

where P is the pressure, $1/C_1$ and ε_c are relevant constants of powder plasticity and critical porosity for the formation of tablets, respectively. ρ is the tablet density and ρ_{true} is the true density.

The above content is our method for determining the true density, hope to make you satisfied

[1] Z. Li, H. Wang, J. Hou, M. Li, X. Shi, B. Liu, Z. Chen, Q. Liu, Q. Fu, Cylindrical granules in the development of mesalazine solid formulations (Ⅱ): The contribution of high aspect ratio to favorable tabletability, International Journal of Pharmaceutics, 649 (2024) 123665.

[2] C. Sun, A Novel Method for Deriving True Density of Pharmaceutical Solids Including Hydrates and Water-Containing Powders, Journal of Pharmaceutical Sciences, 93 (2004) 646-653.
